# A Mendelian randomization study on the causal relationship between smoking, alcohol consumption, and the development of myopia and astigmatism

**DOI:** 10.1038/s41598-024-52316-9

**Published:** 2024-01-22

**Authors:** Diyao Wei, Huanyan Wang, Ling Huang, Minghui Hou, Hong-Gang Liang, Xiang Shi, Xianghui Wei, Jingrong Li, Liuzhu Gan, Bi Lv, Jiabi Deng, Lulu Qing

**Affiliations:** 1 Department of Ophthalmology, Liuzhou Red Cross Hospital/ Eye Hospital of Liuzhou City, Liuzhou, 545001 China; 2 Department of Pediatrics, Third Affiliated Hospital of Sun Yat-Sen University, Guangzhou, 510630 China

**Keywords:** Health policy, Refractive errors

## Abstract

The influence of environmental factors like smoking and alcohol on myopia and astigmatism is controversial. However, due to ethical concerns, alternative study designs are urgently needed to assess causal inference, as mandatory exposure to cigarettes and alcohol is unethical. Following comprehensive screenings, 326 single nucleotide polymorphisms (SNPs) related to myopia and astigmatism were included in the dataset. To validate the causal association between exposures such as cigarette smoking, alcohol consumption, and coffee intake, and outcomes namely astigmatism and myopia, five regression models were employed. These models encompassed MR-Egger regression, random-effects inverse-variance weighted (IVW), weighted median estimator (WME), weighted model, and simple model. The instrumental variables utilized in these analyses were the aforementioned SNPs. Apply Cochran’s Q test to determine heterogeneity of SNPs; if heterogeneity exists, focus on IVW model results. The IVW model showed a 1.379-fold increase in the risk of astigmatism (OR = 1.379, 95%CI 0.822~2.313, *P* = 0.224) and a 0.963-fold increase in the risk of myopia (OR = 0.963, 95%CI 0.666~1.393, *P* = 0.841) for each unit increase in smoking. For each unit increase in coffee intake, the risk of astigmatism increased 1.610-fold (OR = 1.610, 95%CI 0.444~5.835, *P* = 0.469) and the risk of myopia increased 0.788-fold (OR = 0.788, 95%CI 0.340~1.824, *P* = 0.578). For each additional unit of alcohol consumption, the risk of astigmatism increased by 0.763-fold (OR = 0.763, 95%CI 0.380~1.530, *P* = 0.446), and none of the differences were statistically significant. However, for each unit of alcohol consumption, the risk of myopia increased by 1.597 times, and the difference was statistically significant (OR = 1.597, 95%CI 1.023~2.493, *P* = 0.039). The findings indicate that alcohol consumption is a risk factor for myopia but smoking and coffee intake do not affect its development. Additionally, there is no association between smoking, alcohol consumption, coffee intake, and the risk of astigmatism.

## Introduction

Myopia's global prevalence exhibits a consistent upward trajectory, particularly in East and Southeast Asian countries where its prevalence surpasses the global average^1–3^. More than 60% of adults, meanwhile, are afflicted by astigmatism^4^. For a long time, it has been widely postulated that astigmatism and myopia share a close association^5–7^. Furthermore, myopia is associated with many eye diseases such as retinal detachment, cataract, glaucoma, and macular degeneration, and even permanent vision loss in severe cases. A study in Hong Kong China finds higher incidence rate of glaucoma of myopic individuals seeking corneal refractive surgery^8^. The World Health Organization has listed uncorrected refractive error as one of the leading causes of visual impairment worldwide^9^, and elevated vision health to the level of an international public health issue. An important review summarize several risk factors for myopia including age, genetic or acquired, and environmental factors^10^. For example, many researches agree that increased TSO and reduced NW are protective against myopia development among nonmyopes even the strength of evidence is less because of high heterogeneity and lack of clinical trials with clear definition^11^. The underlying mechanism is that when we engage in outdoor activities, we are exposed to an average outdoor light intensity that is approximately 20 times higher than typical indoor lighting. This strong light exposure increases retinal illumination and stimulates dopamine release, which in turn suppresses the axial growth of the eye^12^. Therefore, the early identification of potential risk factors for myopia and astigmatism and the discovery of their specific pathogenesis are of great practical significance to effectively prevent or delay myopia and other refractive errors^13–16^.

Recent epidemiologic studies suggest that, smoking, alcohol consumption and beverage intake may also play a significant role influencing refractive errors such as myopia. A British study from birth to adult observational study found that myopia was positively associated with maternal smoking in early pregnancy and a similar study was done for astigmatism^17^. There is a similar study on astigmatism ^18^. Nevertheless, a study conducted on the UK ALSPAC database presents a contrasting perspective, suggesting that the act of smoking during pregnancy by grandmothers actually diminishes the prevalence of early-onset myopia^19–21^. The Singapore Myopia Risk Factor Cohort Study (STARS) also suggests that maternal smoking may affect ocular development via nicotinic acetylcholine receptors, thereby reducing myopia in children^22,23^. The cross-sectional reports from different regions and age groups in China also support the view that smoking is beneficial to reducing myopia^24–26^. The Spanish prospective cohort study (SUN project) followed the relationship between alcohol intake and myopia over a 10-year period after graduation, and found that alcohol consumption was significantly associated with the prevalence of myopia^27^. More than half of adults with refractive error in India have a drinking habit^28^. Children with Fetal Alcohol Spectrum Disorder (FASD) are more likely to have visual impairment^29^. In conclusion, potential environmental factors like smoking and alcohol consumption can impact the development of myopia and astigmatism, but the exact causal relationship and relative importance are still subject to controversy. However, most of this evidence comes from traditional observational studies, which are susceptible to confounding, reverse causation, and measurement error. Considering the unethical nature of forced exposure to cigarettes and alcohol, randomized controlled trials, are not applicable in the present issue.

Mendelian randomization (MR) is one of the study designs in genetic epidemiology. It utilizes the random splitting and combining of genetically variant gametes to re-randomize populations, and uses genotype as an instrumental variable to assess causality between risk factors and disease. In theory, MR can minimize the influence of confounding factors, circumvent reverse causality, and provide sufficient statistical validity. In recent times, MR has gained widespread utilization in investigating the causality of risk factors in recurrent diseases, primarily relying on the valuable data obtained from genome-wide association studies (GWAS). This approach has enabled researchers to delve into the causal relationship of these risk factors with greater depth and precision [PNS]^30,31^.

Mendelian randomization (MR) has not been widely employed to comprehensively examine the causal connections between potential risk factors and myopia. In a pioneering manner, we have employed MR to investigate the potential impacts of alcohol consumption, coffee intake, and smoking on myopia. Utilizing data from the Genome-Wide Association Studies (GWAS) database, we have made predictions regarding the prevalence risk of myopia and astigmatism. Our findings indicate a lack of evidence supporting a causal relationship between smoking, coffee intake, and astigmatism. However, it is noteworthy that alcohol consumption emerges as a significant risk factor for the development of myopia.

Data sources.

The information on smoking alcohol consumption and coffee intake was obtained through the IEU Open GWAS project (https://gwas.mrcieu.ac.uk/), UK Biobank (http://www.nealelab.is/uk-biobank), FinnGen (https://www.finngen.fi/fi) websites for smoking, alcohol intake frequency, coffee intake, astigmatism and myopia in GWAS data. Site accessed on 2023-08-20.

The final population sources of the data obtained were all European populations, both sexes, whose summary information is presented in Table 1. Relevant data were obtained from five different published GWAS databases, and the original studies had obtained the study Informed consent was obtained from the subjects for the original study and therefore this part of the study did not involve the need for ethics committee approval.

**Table 1:** Summary information on the GWAS database in the MR study.

Variant	Sample size (example)	SNP (example)	Population	Comprehensive database	Gender	Year
Smoking	607,291	11,802,365	European	IEU Open GWAS project	Male and female	2010
Alcohol intake frequency	462,346	9,851,867	European	UK Biobank	Male and female	2018
Coffee intake	428,860	9,851,867	European	UK Biobank	Male and female	2018
Astigmatism	211,588	16,380,455	European	FinnGen	Male and female	2021
Myopia	212,571	16,380,455	European	FinnGen	Male and female	2021

## Methods

**Figure 1:**
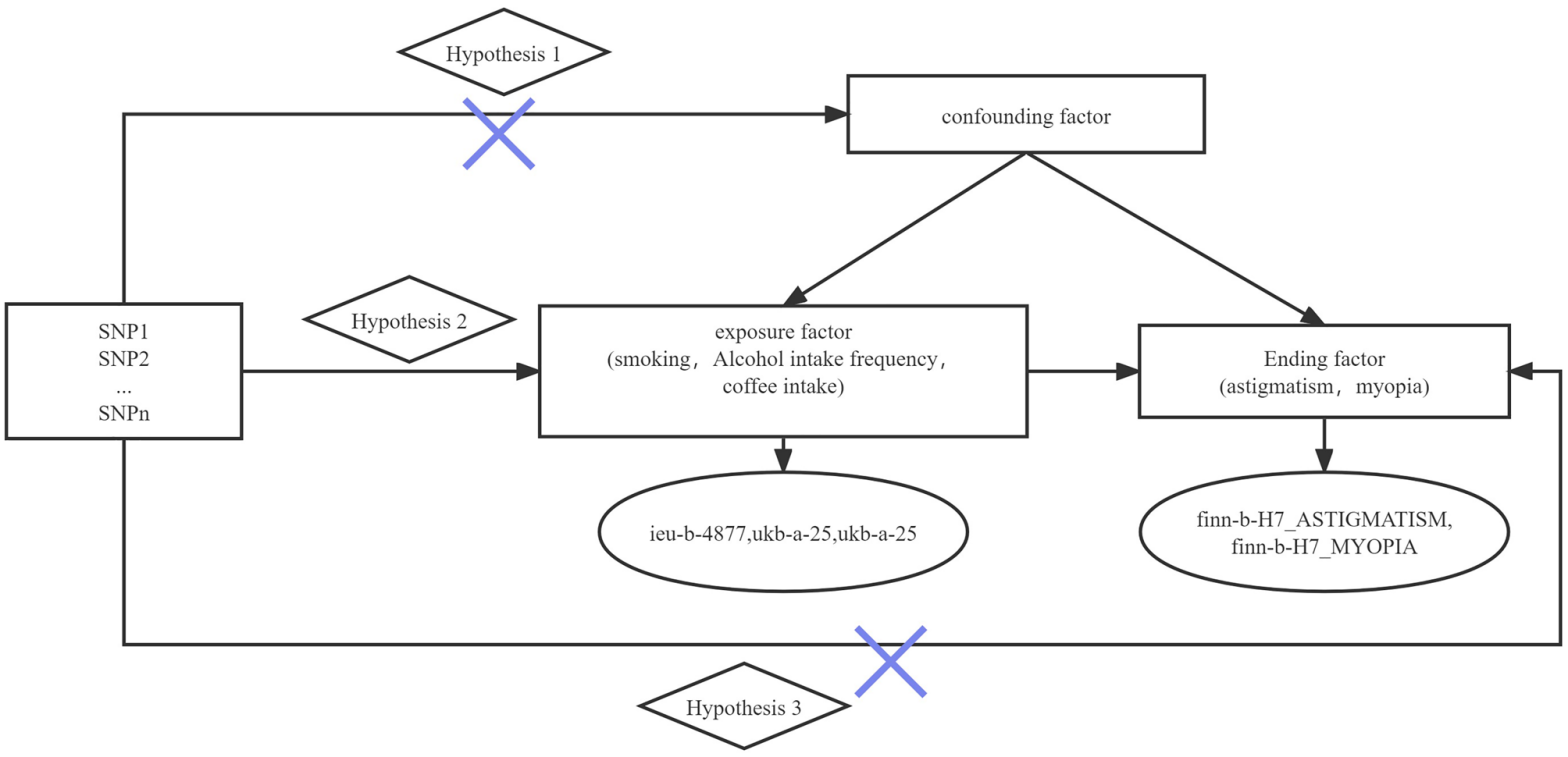
Schematic of Mendelian randomization of the association between smoking and astigmatism. Hypothesis 1: Genotypes must be associated with the exposure factor to be studied; Hypothesis 2: Genotypes must be independent of confounders; Hypothesis 3: Genotypes can only be associated with outcomes by influencing the exposure factor to be studied. ieu-b-4877, ukb-a-25, ukb-b-5237, finn-b-H7_ASTIGMATISM and finn-b-H7_MYOPIA of GWAS were each five published GWAS studies.

The following criteria were used to screen instrumental variables in this study (Fig. 1):

- ①Instrumental variables were highly correlated with exposure, with *P* < 5 × 10^−8^ as the criterion for strong correlation (assumption of association).
- ② The instrumental variables were not directly related to the outcome, but only affected the outcome through exposure, i.e., there was no genetic pleiotropy (exclusivity hypothesis). The absence of genetic pleiotropy is now indicated by a non-zero intercept term in the MR-Egger regression model (*P* > 0.05).
- ③ Instrumental variables must be independent of confounders (independence assumption). Since the SNPs selected by the MR method follow the genetic principle of random assignment of parental alleles to offspring, they are subjected to very little environmental and acquired life, i.e., theoretically, the instrumental variables can be assumed to be independent of environmental factors, such as socio-economic and cultural factors. In addition, the F-statistic greater than 10 was used as an indicator for evaluating weak instrumental variables^32^.

### SNP screening

Significant SNPs were screened from the smoking GWAS pooled data (with *P* < 5 × 10^−8^ as the screening condition, Hypothesis 1); the chain disequilibrium coefficient r 2 was set to be 0.001, and the width of chain disequilibrium region was set to be 10,000 kb to ensure that individual SNPs were independent of each other and to exclude the influence of gene pleiotropy on the results; SNPs associated with confounding factors and endpoints were excluded by the Pheno Scanner associated SNPs (hypotheses 2 and 3). The relevant SNPs screened above were extracted from the GWAS pooled data of astigmatism; a minimum r 2 > 0.8 was set. Information of the above dataset was summarized, while SNPs directly related to astigmatism were excluded (*P* < 5 × 10^−8^).Including the results of the main analysis, heterogeneity analysis and sensitivity analysis.

Five regression models, MR-Egger regression, random-effects inverse-variance weighted (IVW), weighted median estimator (WME), weighted model, and simple model, were used to validate the causal relationship between exposures (smoking, alcohol consumption, and coffee intake) and outcomes (astigmatism, myopia) using SNP as an instrumental variable. We used SNP as an instrumental variable to verify the causal relationship between exposure (smoking, alcohol consumption, coffee intake) and outcome (astigmatism, myopia). The IVW method does not require individual-level data and can directly calculate causal effect values using pooled data, while the MR-Egger regression calculates the correlation between each SNP and astigmatism and myopia (Y) and the correlation between each SNP and smoking, alcohol, and coffee intake (X), and then fits a linear function. The WME method calculates the causal effect estimate of the exposure-endpoint for the jth SNP (βj). Cochran's Q test was applied to determine the heterogeneity of the SNPs; if heterogeneity existed, the IVW model results were focused on; multivariate analyses were performed using the intercept term of the MR-Egger method and the Leave-one-out test; and finally, multivariate MR analyses were used for corrective analyses to adjust for potential confounders. All of the above methods were implemented using the TwoSample MR package in the R 4.2.2 software with a test level of α = 0.05.

## Results

After several screenings, 326 SNPs were finally included in the myopia and astigmatism dataset, and the basic information of some SNPs is shown in Table 2, and the unlisted parts are similar to them. The distribution of F-statistics corresponding to single SNPs ranged from 29.75 to 646.73 (mean 54.29), indicating that causal associations were less likely to be affected by weak instrumental variables.

**Table 2:** Basic information information of some SNPs associated with myopia and astigmatism.

SNP	CHR	POS	EA/OA	EAF	β*	*SE*	*P*
rs13135092	4	103,198,082	G/A	0.083	0.05	0.006	1.48E-14
rs13135092	4	103,198,082	G/A	0.083	0.05	0.006	1.48E-14
rs2472297	15	75,027,880	T/C	0.263	0.046	0.002	1.10E-142
rs2472297	15	75,027,880	T/C	0.263	0.046	0.002	1.10E-142
rs7938812	11	112,911,004	G/T	0.424	0.044	0.004	2.71E-33
rs7938812	11	112,911,004	G/T	0.424	0.044	0.004	2.71E-33
rs12356821	10	104,563,808	C/G	0.14	0.039	0.005	6.27E-15
rs12356821	10	104,563,808	C/G	0.14	0.039	0.005	6.27E-15
rs4410790	7	17,284,577	C/T	0.632	0.039	0.002	1.20E-120
rs4410790	7	17,284,577	C/T	0.632	0.039	0.002	1.20E-120
…	…	…	…	…	…	…	…
rs1260326	2	27,730,940	C/T	0.607	− 0.048	0.004	7.60E-40
rs1260326	2	27,730,940	C/T	0.607	− 0.048	0.004	7.60E-40
rs76608582	19	4,474,725	A/C	0.039	− 0.05	0.008	1.94E-09
rs76608582	19	4,474,725	A/C	0.039	− 0.05	0.008	1.94E-09
rs62305780	4	100,290,815	G/C	0.102	− 0.053	0.006	5.87E-19
rs62305780	4	100,290,815	G/C	0.102	− 0.053	0.006	5.87E-19
rs34805485	9	71,182,471	A/G	0.014	− 0.083	0.015	2.72E-08
rs34805485	9	71,182,471	A/G	0.014	− 0.083	0.015	2.72E-08
rs1229984	4	100,239,319	C/T	0.978	− 0.282	0.012	3.56E-122
rs1229984	4	100,239,319	C/T	0 978	− 0 282	0 012	3 56E-122

### Results

#### Causality verification

**Figure 2:**
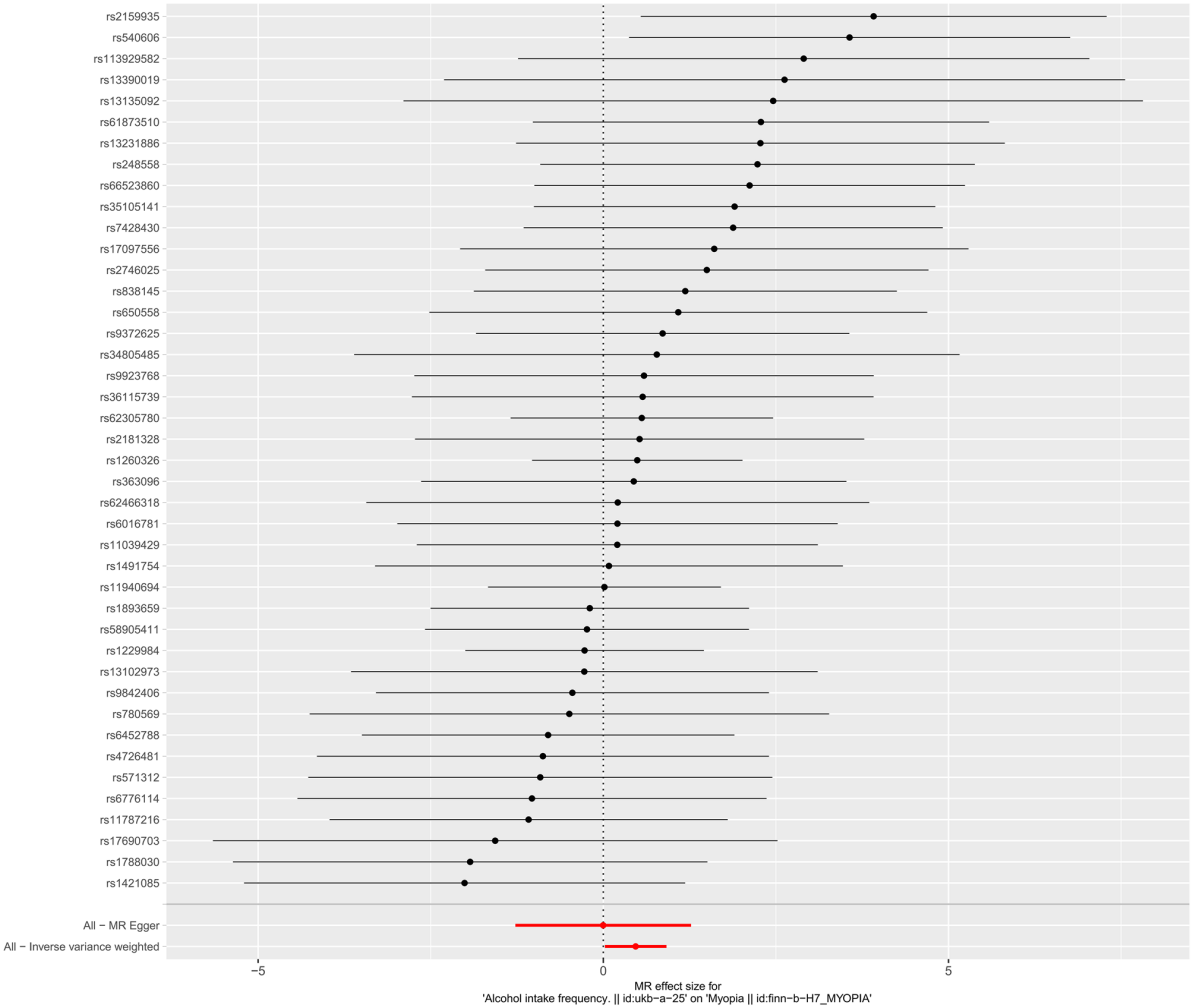
Forest plot of MR findings (alcohol intake-myopia).

Regression results (Table 3), the results suggest that alcohol consumption is a risk factor for the development of myopia, while smoking and coffee intake do not affect the development of myopia, and smoking, alcohol consumption, and coffee intake are not associated with the risk of astigmatism (*P* > 0.05). The IVW model showed that the risk of astigmatism increased by 1.379 times for each unit increase in smoking, but the difference was not statistically significant (OR = 1.379, 95%CI 0.822–2.313, *P* = 0.224), and the results of the MR-Egger regression, WME analysis, weighted model, and simple modeling analysis also showed that there was no direction of causality effect, and the direction of the above five methods was consistent. However, the results of IVW method suggested that the risk of myopia increased by 1.597 times for each unit increase in alcohol consumption, and the difference was statistically significant (OR = 1.597, 95%CI 1.023–2.493, *P* = 0.039), whereas the results of MR-Egger regression, WME analysis, weighted model, and simple model showed no statistically significant direction, and the direction of causality was inconsistent, taking the results of IVW method as a whole. The results of IVW method were taken in synthesis (Fig. 2).

**Table 3:** Results of causal association of 5 methods MR regression.

Exposure factor	Ending variable	SNP	Method	β	SE	OR (95%CI)	*P*
Smoking	Astigmatism	83	MR-Egger regression WME Analysis IVW models simple model weighting model	0.271 − 0.261 0.321 − 0.862 − 0 704	1.322 0.372 0.264 0.892 0 791	1.311 (0.098~17.506) 0.770 (0.372~1.596) 1.379(0.822~2.313) 0.422(0.074~2.425) 0 495 (0 105~2 332)	0.838 0.482 0.224 0.337 0 376
Smoking	Myopia	83	MR-Egger regression WME Analysis IVW models simple model weighting model	− 1.584 − 0.072 − 0.038 0.171 0 364	0.933 0.246 0.188 0.704 0 737	0.205 (0.033~1.277) 0.931 (0.575~1.506) 0.963 (0.666~1.393) 1. 187(0.299~4.712) 1 439(0 340~6 099)	0.093 0.770 0.841 0.808 0 622
Alcohol intake Frequency	Myopia	42	MR-Egger regression WME Analysis IVW models simple model weighting model	0.532 − 0.352 − 0.270 − 0.737 − 0.463	1.011 0.530 0.355 0.994 0.853	1.702 (0.235~12.342) 0.703 (0.249~1.986) 0.763 (0.380~1.530) 0.478 (0.068~3.357) 0.629 (0.118~3.351)	0.602 0.506 0.446 0.462 0.590
Alcohol intake Frequency	Myopia	42	MR-Egger regression WME Analysis IVW models simple model weighting model	− 0.002 0.327 0.468 0.191 0.135	0.648 0.318 0.227 0.588 0.467	0.998 (0.280~3.556) 1.387 (0.743~2.589) 1.597 (1.023~2.493) 1.211 (0.382~3.835) 1. 144 (0.459~2.857)	0.997 0.304 0.039 0.747 0.774
Coffee intake	Astigmatism	38	MR-Egger regression WME Analysis IVW models simple model weighting model	0.684 0.341 0.476 − 0.239 0.105	1.339 0.906 0.657 1.747 0.932	1.983 (0.144~27.358) 1.407 (0.238~8.313) 1.610 (0.444~5.835) 0.787 (0.026~24.154) 1. 111 (0.179~6.905)	0.612 0.706 0.469 0.892 0.911
Coffee intake	Myopia	38	MR-Egger regression WME Analysis IVW models simple model weighting model	0.118 − 0.433 − 0.238 − 1.020 − 0.538	0.871 0.617 0.428 1.256 0.666	1.125 (0.204~6.198) 0.648 (0.193~2.175) 0.788 (0.340~1.824) 0.361 (0.031~4.224) 0.584 (0.158~2.152)	0.893 0.483 0.578 0.422 0.424

#### Heterogeneity test

**Figure 3:**
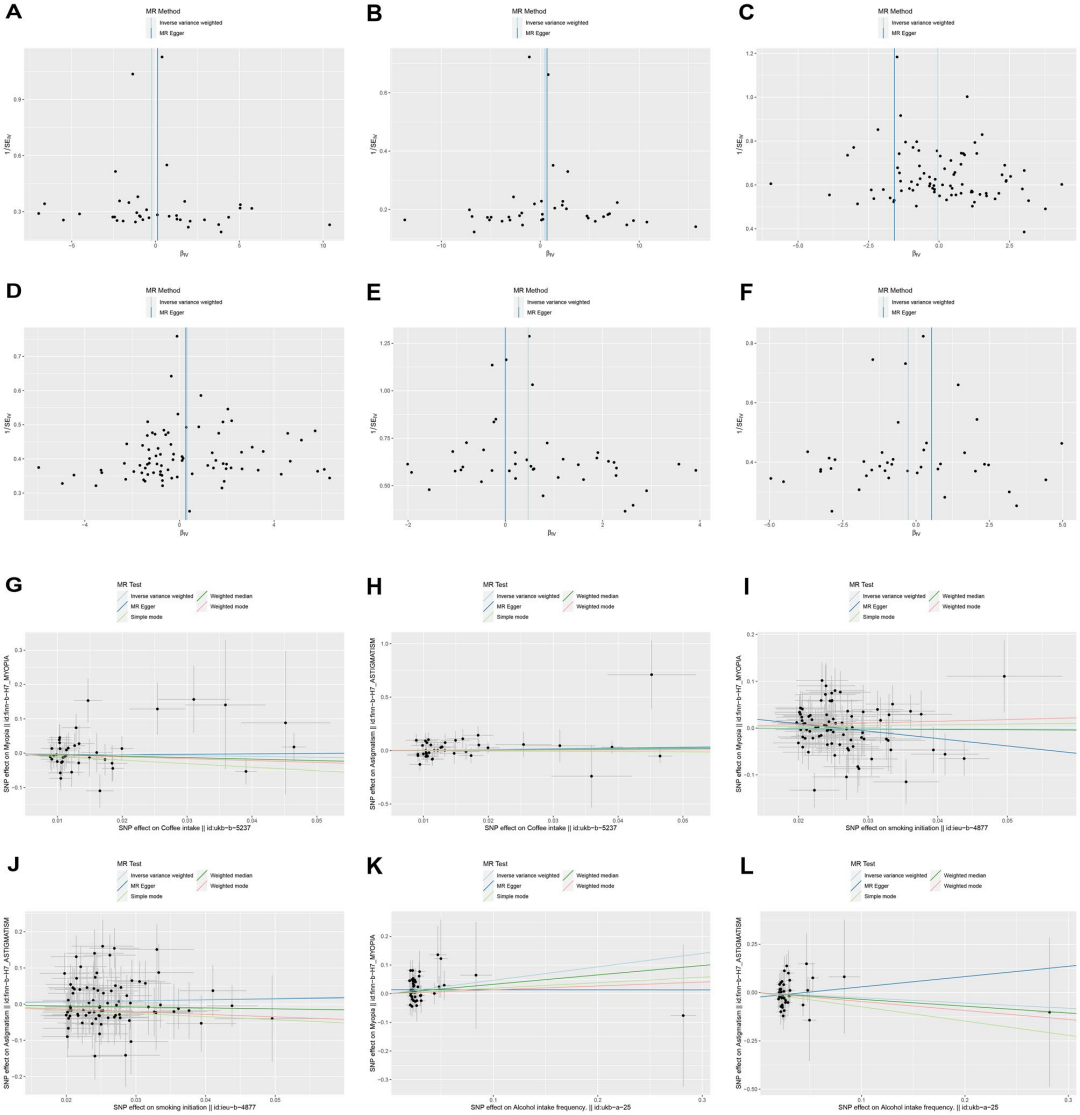
funnel plot (**A-F**) and Scatter plot (**G-L**) of Mendelian randomization.

The statistic Q shown by MR-Egger regression and Cochran Q test of IVW method, both *P* > 0.05, suggested that there was no heterogeneity among SNPs (Table 4). And the difference between the MR-Egger regression intercept term and 0 was not statistically significant (*P* > 0.05), so we concluded that there was no genetic pleiotropy among the SNPs (Table 5). The scatterplot and funnel plot showed that the distributions of all the included SNPs were largely symmetrical, suggesting that causal associations were less likely to be affected by potential bias (Fig. 3).

**Table 4:** MR heterogeneity analysis results.

Exposure factor	Ending variable	Method	StatisticsQ	*P*
Smoking	Astigmatism	MR-Egger regression	76.256	0.628
		IVW models	76.257	0.658
	Myopia	MR-Egger regression	98.412	0.091
		IVW models	101.887	0.068
Alcohol intake frequency	Astigmatism	MR-Egger regression	32.550	0.793
		IVW models	33.268	0.799
	Myopia	MR-Egger regression	28.805	0.906
		IVW models	29.405	0.912
Coffee intake	Astigmatism	MR-Egger regression	36.998	0.423
		IVW models	37.031	0.468
	Myopia	MR-Egger regression	38.161	0.371
		IVW models	38.397	0.406

**Table 5:** MR-Egger regression analysis of instrumental variables.

exposure factor	Ending variable	Intercept	*SE*	*P*
Smoking	Astigmatism	0.001	0.034	0.969
Smoking	Myopia	0.041	0.024	0.095
Alcohol intake frequency	Astigmatism	− 0.024	0.028	0.402
Alcohol intake frequency	Myopia	0.014	0.018	0.443
Coffee intake	Astigmatism	− 0.004	0.022	0.859
Coffee intake	Myopia	− 0.007	0.014	0.640

#### Sensitivity analysis

**Figure 4:**
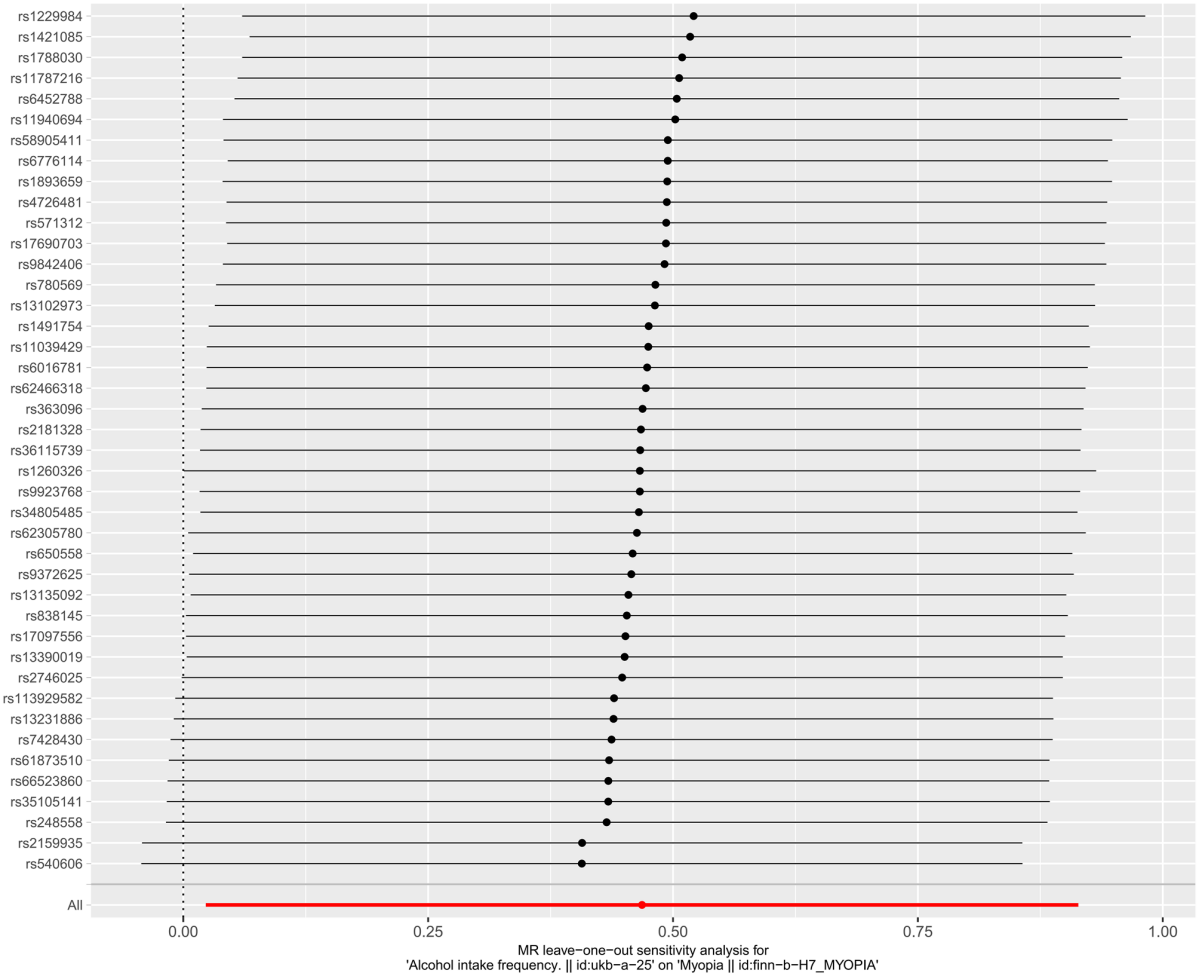
Results of "Leave-one-out" sensitivity analysis.

After the Leave-one-out test, the analysis results of the remaining SNPs were similar to those of the inclusion of all SNPs after removing each SNP of smoking in turn, and no SNPs were found to have a large impact on the causal association estimates, indicating that the MR results of this study were robust (Fig. 4).

## Discussion

In this study, we used a sample of studies from the GWAS database to predict the prevalence risk of myopia and astigmatism. We found no evidence of a causal relationship between smoking, coffee intake and astigmatism. However, the results of IVW method suggested that the risk of myopia prevalence increased by 1.597 times for each unit increase in alcohol consumption, and the difference was statistically significant (OR = 1.597, 95%CI 1.023~2.493, *P* = 0.039), while in the SUN cohort study Alcohol intake was linearly and significantly associated with a higher risk of myopia development or progression with the OR for 10-year incidence/progression of myopia was 1.05, 95% CI 1.01–1.09 per each 10-g increase in alcohol intake^27^.

Mendelian randomized causal inference is commonly used in MR-Egger regression, inverse-variance weighted (IVW), weighted median estimator (WME), weighted model, simple model, etc. Among them, IVW method does not require individual-level data and can use pooled data to calculate causal effect values directly, which is the main analysis method. Among them, the IVW method does not require individual-level data and can directly calculate causal effect values using aggregated data, which is the main analysis method, while the MR-Egger regression and the WME method are supplementary analysis methods. In the causal relationship between alcohol intake and myopia, the IVW method suggested that the difference between the results was statistically significant, and subsequent sensitivity analyses suggested that the present results were robust, i.e., the conclusion that alcohol intake is a risk factor for myopia was valid although the results of the MR-Egger regression, the WME analysis, the weighted model, and the simple model showed no statistically significant difference.

We speculate that there may be the following mechanisms: (1) Dopamine regulation: Alcohol consumption has been shown to decrease dopamine secretion in the brain. As dopamine plays a role in regulating eye growth and preventing myopia, the reduction in dopamine levels caused by alcohol may disrupt this regulatory process. (2) Visual processing impairment: Alcohol can impair visual processing and perception. This may lead to difficulties in focusing and strain on the eyes, potentially contributing to the development or progression of myopia. (3) Lifestyle factors: Alcohol consumption is often associated with certain lifestyle habits, such as spending less time outdoors and engaging in more sedentary activities. These factors have been linked to an increased risk of myopia. (4) Nutritional deficiencies: Excessive alcohol consumption can lead to nutritional deficiencies, such as deficiencies in vitamins and minerals that are essential for eye health. These deficiencies may contribute to the development of myopia. However, it is important to note that these are speculative mechanisms and further research is needed to confirm their validity.

In summary, this study further illustrates the causal relationship between alcohol consumption and myopia, which is useful for the promotion of myopia prevention and control. However, the results of the analysis show that the results of alcohol consumption and myopia are not completely unified, and the results of the IVW method, as well as the individual differences between people and other living habits, need to be further investigated.

## Data Availability

The datasets analyzed during the current study are available in the IEU Open GWAS project (https://gwas.mrcieu.ac.uk/), UK Biobank (http://www.nealelab.is/uk-biobank), FinnGen (https://www.finngen.fi/fi).
